# Factors Associated with Uninterpretable Spirometry During Asthma Screening in Children with Sickle Cell Disease: An Expanded Multicenter Study in French Guiana

**DOI:** 10.3390/children13091281

**Published:** 2026-09-21

**Authors:** Gabriel Bafunyembaka, Joddy Mafema, Christian Kinsiona, Nadia Nathan, Narcisse Elenga

**Affiliations:** 1 Department of Pediatrics, University Hospital of Guiana, Saint-Laurent-du-Maroni Site, 1465 Boulevard de la Liberté, 97320 Saint-Laurent-du-Maroni, French Guiana, France; 2 Department of Pediatrics, Provincial General Reference Hospital of Bukavu, 2 Avenue Michombero, Bukavu, South Kivu, Democratic Republic of the Congo; 3 Department of Pediatrics, Cayenne Hospital, 3 Avenue Alexis Blaise, 97300 Cayenne, French Guiana, France; joddy.mafemamissindu@ch-cayenne.fr (J.M.); narcisse.elenga@ch-cayenne.fr (N.E.); 4 Department of Pediatrics, Clinique La Canopée, 5 Avenue de l’Université, Harvard Hibiscus Business District, 97300 Cayenne, French Guiana, France; christian.kinsiona@cliniquecanopee-rainbow.com; 5 Pediatric Pulmonology Department, Armand Trousseau Hospital, Assistance Publique–Hôpitaux de Paris (AP-HP), Sorbonne Université, 26 Avenue du Dr Arnold Netter, 75012 Paris, Île-de-France, France; nadia.nathan@aphp.fr

**Keywords:** sickle cell disease, spirometry, asthma screening, children, pulmonary function testing, French Guiana, test interpretability

## Abstract

**Highlights:**

**What are the main findings?**

- Technically uninterpretable spirometry occurred in 23.8% of children undergoing systematic asthma screening.
- Younger age was associated with a higher likelihood of an uninterpretable examination in univariable analysis.

**What are the implications of the main findings?**

- Age-adapted preparation, experienced coaching, and planned repeat testing should be prioritized for younger children.
- Harmonized spirometry quality-control procedures across centers may improve screening yield and reduce missed respiratory disease.

**Abstract:**

Background/Objectives: Spirometry is essential for objective asthma assessment in children with sickle cell disease (SCD), but technically uninterpretable examinations may reduce the effectiveness of systematic screening. This study aimed to determine their frequency and identify associated demographic, clinical, social, disease-related, and center-level factors in French Guiana. Methods: We conducted a prospective multicenter observational study involving children and adolescents with confirmed SCD who underwent systematic asthma screening at Cayenne, Kourou, and Saint-Laurent-du-Maroni hospitals between January 2025 and May 2026. Spirometry technical quality was assessed according to the 2019 American Thoracic Society/European Respiratory Society technical standards and classified as interpretable or technically uninterpretable according to the acceptability, usability, and repeatability of the recorded maneuvers. Across the three sites, spirometry was performed on the recorded MicroLab platform using GLI-2012 reference equations. Salbutamol was delivered by pressurized metered-dose inhaler with a valved holding chamber when bronchodilator testing was technically feasible; the exact device submodel/manufacturer metadata, center-specific software versions, and administered salbutamol dose were not retained in the exported analytical dataset. Participant characteristics were compared between groups, and associated factors were examined using univariable and multivariable logistic regression. Results: After removal of duplicate records, 143 unique children were included. Spirometry was technically uninterpretable in 34 participants (23.8%). Children with uninterpretable examinations were significantly younger than those with interpretable spirometry (8.9 ± 3.0 vs. 10.5 ± 4.0 years; *p* = 0.015). In univariable analysis, each additional year of age was associated with a 12% reduction in the odds of uninterpretable spirometry (OR 0.88, 95% CI 0.78–0.99; *p* = 0.036). After adjustment for sex, recruitment center, and health-insurance status, the inverse association persisted in direction and magnitude but did not reach conventional statistical significance (adjusted OR 0.89, 95% CI 0.79–1.01; *p* = 0.075). Given the limited number of outcome events, this attenuation may reflect reduced statistical power. No other investigated factor was independently associated with non-interpretability. Conclusions: Nearly one-quarter of children had technically uninterpretable spirometry. Younger children may benefit from enhanced preparation, age-adapted coaching, experienced operators, and planned repeat testing to improve asthma-screening yield and reduce missed respiratory disease.

## 1. Introduction

Sickle cell disease (SCD) is one of the most common inherited monogenic disorders worldwide and remains a major cause of childhood morbidity, premature mortality and healthcare utilisation. Recent Global Burden of Disease estimates indicate that the health burden attributable to SCD is substantially greater than previously recognised, particularly in populations of African ancestry and in regions where access to comprehensive care remains unequal [1]. Although vaso-occlusion and chronic haemolysis are central to its pathophysiology, respiratory complications contribute considerably to disease severity from childhood onwards. These include acute chest syndrome, recurrent wheezing, hypoxaemia, airway obstruction, impaired lung growth and progressive abnormalities in pulmonary function [2,3].

Acute chest syndrome is one of the most severe pulmonary complications of SCD and a major cause of hospitalisation and acute respiratory deterioration in affected children [2]. Its clinical manifestations—including cough, wheezing, dyspnoea, chest pain and hypoxaemia—may overlap with those of asthma, complicating the recognition of chronic airway disease. Respiratory symptoms may be attributed solely to SCD, infection or previous acute chest syndrome, while SCD-related pulmonary abnormalities may conversely be interpreted as asthma without adequate objective confirmation. This distinction is clinically important because asthma represents a potentially modifiable comorbidity associated with increased respiratory morbidity. A recent systematic review and meta-analysis found that patients with both SCD and asthma had a substantially increased risk of acute chest syndrome [4]. In French Guiana, asthma was similarly identified as an independent risk factor for acute chest syndrome among children with SCD [5].

Pulmonary-function abnormalities are not restricted to children with a previously recognised asthma diagnosis. Studies in children and adolescents with SCD have reported reduced forced expiratory volume in one second (FEV_1_), forced vital capacity (FVC), FEV_1_/FVC ratio and lung volumes, as well as obstructive, restrictive and mixed ventilatory patterns [3,6,7,8,9]. Longitudinal investigations suggest that abnormal respiratory trajectories may develop during childhood, with attenuated growth or progressive decline in lung-function indices as patients become older [6,7,8,9]. These findings support the integration of objective respiratory assessment into routine SCD care, including for children without an established respiratory diagnosis.

International asthma guidelines recommend that diagnosis in school-aged children should be based on a history of variable respiratory symptoms together with objective evidence of variable expiratory airflow limitation whenever testing is available [10]. Spirometry with bronchodilator reversibility testing therefore plays a central role in the investigation of suspected asthma. It enables the measurement of FEV_1_, FVC and the FEV_1_/FVC ratio, the identification of airflow obstruction and the assessment of functional improvement after bronchodilator administration [10,11,12]. In children with SCD, spirometry is particularly valuable because symptoms and clinical questionnaires alone may not reliably distinguish asthma from other SCD-related respiratory manifestations.

However, the diagnostic value of spirometry depends fundamentally on the technical quality of the recorded manoeuvres. Spirometry is an effort-dependent examination requiring the child to understand the instructions, achieve a maximal inspiration, initiate expiration rapidly, maintain a forceful expiratory effort and repeat the manoeuvre consistently. The 2019 American Thoracic Society/European Respiratory Society technical standard defines acceptability, usability and repeatability criteria that should be assessed before physiological interpretation [11]. Common causes of technically inadequate tests include incomplete inspiration, hesitation at the start of expiration, cough, glottic closure, air leakage, variable effort, premature termination and failure to obtain sufficiently repeatable FEV_1_ or FVC measurements [11]. When these criteria are not met, the examination may not reliably establish normal lung function, airflow obstruction or bronchodilator responsiveness. A technically uninterpretable examination must therefore not be considered equivalent to a normal result or to the absence of asthma [11,12].

Although most school-aged children are able to perform spirometry, test success is influenced by developmental, patient-related and procedural factors. Age is one of the most consistently documented determinants of acceptable and repeatable spirometry, with younger children generally showing higher rates of technically unsuccessful examinations [13]. Nevertheless, clinically useful spirometry can be obtained in many young children when age-adapted explanations, visual encouragement, experienced coaching and repeated attempts are provided [14]. These findings suggest that non-interpretability is not determined by age alone and may also reflect modifiable factors related to preparation, testing conditions, operator experience and local organisation.

These issues are particularly relevant when spirometry is implemented as part of a systematic screening programme rather than being restricted to specialised paediatric pulmonary-function laboratories. Screening programmes involve children with heterogeneous clinical characteristics, varying familiarity with respiratory testing and different capacities to understand and reproduce complex manoeuvres. In multicentre settings, differences in spirometry success may also reflect variation in equipment, appointment duration, operator training, coaching methods and application of quality-control criteria.

French Guiana represents a distinctive context for such an evaluation. The territory has a high burden of SCD and a geographically dispersed, multicultural and multilingual population. Children with SCD are principally followed in Cayenne, Kourou and Saint-Laurent-du-Maroni, with potential differences in residence, travel distance, access to specialised care and local healthcare organisation. These factors may influence the feasibility of an effort-dependent investigation such as spirometry.

An initial prospective multicentre study conducted in French Guiana between January and July 2025 included 140 children with SCD aged 5–17 years. Systematic screening combined clinical assessment, the International Study of Asthma and Allergies in Childhood questionnaire, and spirometry with bronchodilator reversibility testing. Spirometry-confirmed asthma was identified in 30.7% of participants, while 22.7% of spirometric tracings were non-interpretable, leaving approximately one-fifth of the children without a definitive functional classification [15]. The initial study primarily assessed asthma prevalence and the diagnostic performance of the screening strategy and did not specifically investigate the factors associated with spirometry non-interpretability.

The subsequent extension of the programme from January 2025 to May 2026 across the same three hospital centres provides an opportunity to address this unresolved question in a larger cohort. Identifying factors associated with uninterpretable spirometry could help recognise children requiring enhanced preparation, longer appointments, experienced operators, repeated testing or alternative respiratory investigations. It could also reveal potentially modifiable centre-level differences and reduce the risk of incorrectly considering technically unsuccessful examinations as normal.

However, factors associated with technically uninterpretable spirometry during systematic asthma screening in children with SCD remain insufficiently characterized, particularly in geographically dispersed and multilingual multicenter settings. Therefore, this study aimed to determine the frequency of technically uninterpretable spirometry and to identify demographic, clinical, disease-related, geographic, social, and center-related factors associated with spirometry non-interpretability among children with SCD undergoing systematic asthma screening in French Guiana.

## 2. Materials and Methods

### 2.1. Study Design and Setting

This prospective multicenter observational study was conducted using an expanded cohort of children and adolescents with sickle cell disease who underwent systematic respiratory assessment in French Guiana. Participants were recruited between January 2025 and May 2026 at the three principal hospitals providing pediatric sickle cell disease care in the territory: Cayenne Hospital, Kourou Hospital, and the Saint-Laurent-du-Maroni site of the University Hospital of French Guiana.

The expanded cohort included children enrolled in the initial asthma-screening study conducted between January and July 2025, together with additional participants subsequently assessed through May 2026 using the same screening pathway. The present study addressed a distinct research question from the initial publication and focused specifically on factors associated with technically uninterpretable spirometry.

### 2.2. Study Population

Children and adolescents were eligible if they had a confirmed diagnosis of sickle cell disease, were followed at one of the participating hospitals, and underwent respiratory screening during the study period.

All major sickle cell genotypes were eligible, including HbSS, HbSC, HbS/β^0^-thalassemia, and HbS/β^+^-thalassemia. Respiratory assessment was performed when participants were clinically stable and outside an acute vaso-occlusive crisis, acute chest syndrome, febrile illness, or acute respiratory exacerbation.

Participants were excluded from the primary analysis when no spirometry attempt had been performed, when the spirometry report was unavailable, or when the available information was insufficient to determine whether the examination was technically interpretable.

Only one observation per participant was retained in the analytical dataset. When repeated assessments were identified, the first complete screening assessment meeting the eligibility criteria was selected, unless the subsequent assessment contained the only available technically documented spirometry result.

### 2.3. Respiratory Screening Pathway

Respiratory screening was integrated into routine pediatric sickle cell disease follow-up. The standardized evaluation included review of respiratory symptoms, previous respiratory diagnoses, history of wheezing, exercise-related symptoms, nocturnal symptoms, previous episodes of acute chest syndrome, and prior clinical suspicion or diagnosis of asthma.

The screening pathway also included completion of the International Study of Asthma and Allergies in Childhood questionnaire, a standardized clinical examination, baseline spirometry, and bronchodilator reversibility testing when technically feasible.

Allergy skin-prick testing, chest radiography, and stool parasitology were performed within the broader screening programme when indicated or available. These investigations were not used to define the technical interpretability of spirometry but could be considered as clinical or contextual covariates.

### 2.4. Spirometry Procedure

Spirometry was performed by trained healthcare professionals using standardized pediatric procedures and common technical quality criteria consistent with the 2019 American Thoracic Society/European Respiratory Society recommendations [11]. Age-adapted explanations and real-time coaching were used at all three participating centers. Although common quality criteria were applied, standardized operator-level identifiers and measures of operator experience were not available for separate analysis.

Before testing, the procedure was explained to each participant using age-adapted instructions. Children were asked to perform a maximal inspiration followed by a rapid, forceful, and sustained expiration. Several attempts were permitted to obtain technically acceptable and reproducible flow–volume curves.

The principal spirometric variables assessed were forced expiratory volume in one second (FEV1), forced vital capacity (FVC), and the FEV1/FVC ratio. Spirometric values were expressed using the Global Lung Function Initiative 2012 [16] (GLI-2012) reference equations. The spirometer software automatically calculated predicted values and z-scores; the software version was not retained in the exported analytical dataset.

Technical quality was evaluated according to the acceptability, usability, and repeatability of the recorded maneuvers. Particular attention was paid to incomplete inspiration, excessive hesitation at the start of expiration, cough during the maneuver, glottic closure, air leakage, submaximal effort, variable effort, premature termination, and insufficient repeatability between attempts.

Bronchodilator reversibility testing was performed when baseline spirometry was technically adequate. Salbutamol was administered through a pressurized metered-dose inhaler with a valved holding chamber, and post-bronchodilator spirometry was repeated after approximately 15 min. The administered salbutamol dose was not retained in the exported analytical dataset and is therefore not reported. A response was considered positive when FEV1 increased by at least 12% from the baseline value.

Spirometry was performed using MicroLab spirometers in the participating hospitals. The same standardized pediatric testing and quality-control procedures were applied at Cayenne, Kourou, and Saint-Laurent-du-Maroni, and GLI-2012 reference equations were used across sites. The archived analytical dataset retained the MicroLab device family but not the exact device submodel, manufacturer metadata, or center-specific software versions; these details therefore cannot be reported reliably by individual site.

### 2.5. Primary Outcome

The primary outcome was technically uninterpretable spirometry.

Spirometry was classified as interpretable when the available maneuvers were of sufficient technical quality to permit reliable assessment of forced expiratory volume in one second, forced vital capacity, and the forced expiratory volume in one second to forced vital capacity ratio, irrespective of whether the physiological result was normal or abnormal.

Spirometry was classified as uninterpretable when the quality of the examination was insufficient to permit reliable physiological interpretation. This included examinations with no acceptable maneuver, insufficient repeatability, incomplete inspiration, excessive hesitation, cough or glottic closure affecting measurement, premature termination, persistently submaximal or inconsistent effort, or technically inadequate flow–volume curves.

An uninterpretable examination was not considered equivalent to normal spirometry and was not used to exclude asthma.

When the initial database classification was incomplete or contradictory, the original spirometry report and, when available, the flow–volume curves were reviewed. Participants whose spirometry interpretability remained uncertain after source-data review were classified as having an indeterminate technical status and were excluded from the primary binary analysis.

### 2.6. Reconstruction of Spirometry Interpretability

A new mutually exclusive variable was created to classify participants as having interpretable, uninterpretable, or indeterminate spirometry.

This reconstructed variable was based primarily on the technical quality assessment documented in the spirometry report rather than on the pre-existing diagnostic variables for confirmed asthma, absence of asthma, or unclassified respiratory status.

The hierarchy used for reconstruction was as follows:

- Examinations explicitly reported as technically acceptable and allowing physiological interpretation were classified as interpretable;
- Examinations explicitly reported as technically unacceptable, non-reproducible, or non-interpretable were classified as uninterpretable;
- Records with insufficient or contradictory information after source verification were classified as indeterminate.

The original diagnostic variables were retained for traceability but were not used as the primary definition of spirometry interpretability.

### 2.7. Reconstruction of Respiratory Classification

After determination of spirometry quality, a separate mutually exclusive respiratory classification was created.

Participants with technically interpretable spirometry and functional evidence meeting the predefined criteria for airflow obstruction and significant bronchodilator reversibility were classified as having spirometry-supported asthma.

Participants with technically interpretable spirometry who did not meet the predefined functional criteria were classified as not having spirometry-supported asthma. This category did not imply definitive exclusion of asthma, because a single normal or non-reversible spirometry examination may not exclude variable airway disease.

Participants with technically uninterpretable spirometry were not assigned to either the spirometry-supported asthma or no spirometry-supported asthma categories.

Participants with unresolved or contradictory source data were classified separately as indeterminate.

### 2.8. Data Cleaning and Duplicate Identification

Data cleaning was performed before statistical analysis. Variable names, response categories, spelling variants, and missing-value codes were standardized. Binary variables were harmonized to ensure consistent coding.

Potential duplicate records were identified using combinations of available demographic and clinical variables, including date of birth, sex, sickle cell genotype, participating hospital, place of residence, and patterns of clinical and screening information.

Exact duplicate rows were flagged automatically. Records sharing the same date of birth and sex were not automatically removed because these characteristics alone were insufficient to establish that two records represented the same child.

Potential duplicates underwent manual review using all available information. When two records were confirmed to correspond to the same participant, only one record was retained. Priority was given to the most complete record containing the best-documented spirometry quality assessment.

The numbers of records assessed, duplicate records excluded, and unique participants retained were reported in the study flow diagram.

### 2.9. Candidate Explanatory Variables

Potential factors associated with uninterpretable spirometry were selected according to clinical relevance, biological plausibility, and availability in the expanded database.

Demographic and social variables included age, sex, family context, health insurance status, place of residence, and rural or urban origin.

Sickle cell disease-related variables included genotype, hydroxyurea treatment, history of acute chest syndrome, frequency of hospital admissions, and indicators of disease severity.

Respiratory and atopic variables included previous clinical suspicion or diagnosis of asthma, wheezing, exercise-related respiratory symptoms, nocturnal symptoms, rhinitis, eczema, tobacco-smoke exposure, and relevant questionnaire responses.

Organizational variables included the participating hospital and the recruitment period. When available, information concerning the operator, testing location, or equipment was also considered.

Variables that could only be obtained after successful spirometry, such as measured airflow obstruction or bronchodilator reversibility, were not treated as baseline determinants of technical failure.

### 2.10. Statistical Analysis

Participant characteristics were summarized for the overall cohort and according to spirometry interpretability. Normality of continuous variables was assessed using the Shapiro–Wilk test and visual inspection of distributions. Approximately normally distributed variables were presented as means and standard deviations, whereas skewed variables were presented as medians and interquartile ranges. Categorical variables were reported as frequencies and percentages.

Continuous variables will be presented as mean and standard deviation when approximately normally distributed and as median and interquartile range when distributions are skewed. Categorical variables will be reported as frequencies and percentages.

Children with interpretable and uninterpretable spirometry were compared using Student’s *t*-test or the Mann–Whitney U test for continuous variables and Pearson’s chi-square test or Fisher’s exact test for categorical variables, as appropriate.

The proportion of uninterpretable spirometry examinations was reported with its 95% confidence interval.

Univariable logistic regression was used to estimate crude odds ratios and 95% confidence intervals for associations with technically uninterpretable spirometry.

A parsimonious multivariable logistic regression model was constructed with technically uninterpretable spirometry as the dependent variable. Age and sex were included a priori; recruitment center and health-insurance status were retained because of their clinical and contextual relevance. The number of covariates was restricted because only 34 outcome events were observed.

Multicollinearity was assessed using variance inflation factors, and model calibration was evaluated using the Hosmer–Lemeshow goodness-of-fit test. The archived analysis indicated no problematic multicollinearity and no evidence of poor model calibration. However, the numerical VIF values and the Hosmer–Lemeshow test statistic, degrees of freedom, and *p*-value were not preserved in the exported final-analysis output; consequently, exact diagnostic values cannot be reported without re-running the model from the source analytical dataset.

Age was analyzed primarily as a continuous variable and secondarily using clinically relevant pediatric age groups (5–7, 8–11, and 12–18 years).

Associations between participating center and spirometry interpretability were interpreted cautiously because center-level differences may reflect variation in participant characteristics, operator experience, equipment, testing conditions, or completeness of technical documentation.

Missing data were not coded as negative responses. The primary multivariable analysis used complete cases.

All statistical tests were two-sided, and *p* < 0.05 was considered statistically significant. Analyses were performed using Stata, version 17.

### 2.11. Ethical Considerations

The respiratory screening programme was conducted as part of routine clinical assessment of children with SCD in French Guiana. The present study involved secondary analysis of pseudonymized data collected during routine care and was conducted in accordance with the Declaration of Helsinki and applicable French regulations governing research using healthcare data.

No additional research-specific procedure was performed for this analysis. Parents or legal guardians had been informed about the clinical screening pathway and use of pseudonymized clinical data; no additional written consent was required for this secondary analysis, in accordance with the institutional determination stated below.

Data were analyzed in pseudonymized form, and no directly identifying information was included in the analytical dataset.

The institutional review determination was as follows: the study was based exclusively on secondary analysis of pseudonymized routine-care data, with no additional or unusual diagnostic, therapeutic, or monitoring procedure; therefore, additional ethics committee approval was not required under the applicable French framework. This determination was made within the University Hospital of French Guiana institutional framework. Data were processed in accordance with institutional data-protection requirements; no separate ethics committee or protocol reference number was assigned for this secondary analysis.

This manuscript was prepared in accordance with the Strengthening the Reporting of Observational Studies in Epidemiology (STROBE) statement. The completed STROBE checklist is provided as Supplementary Material File S1.

## 3. Results

### 3.1. Participant Flow and Spirometry Interpretability

**Figure 1:**
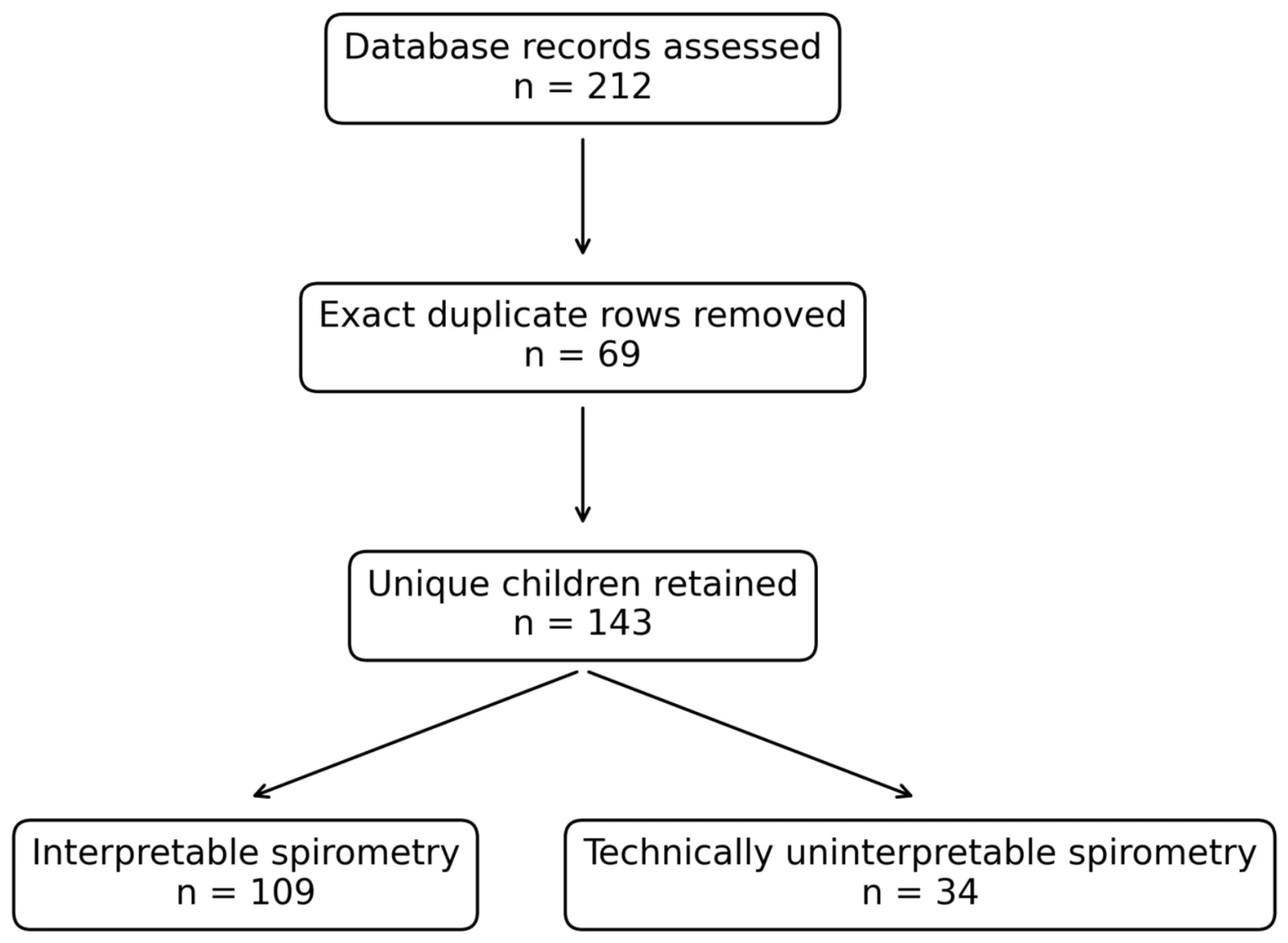
Flow diagram of database cleaning and final spirometry classification.

The source database contained 212 records. After removal of 69 strictly identical duplicate records, 143 unique children were retained in the final analysis. Among them, 34 had technically uninterpretable spirometry, corresponding to 23.8% of the study population, whereas 109 had an interpretable spirometric examination. The participant selection process and final spirometry classification are presented in Figure 1.

### 3.2. Participant Characteristics

The mean age of the study population was 10.2 ± 3.8 years. Children with technically uninterpretable spirometry were significantly younger than those with interpretable spirometry, with a mean age of 8.9 ± 3.0 years compared with 10.5 ± 4.0 years, respectively (*p* = 0.015).

As shown in Table 1, no statistically significant differences were observed between the two groups regarding sex, health-insurance status, sickle cell genotype, clinical severity, rural origin, recruitment centre, hydroxyurea treatment, previous acute chest syndrome, prior asthma suspicion, tobacco-smoke exposure, ISAAC questionnaire responses, eczema, or rhinitis.

Centre-level distribution in the archived analysis output was as follows: at Kourou, 28 of 41 examinations were interpretable (68.3%) and 13 of 41 were technically uninterpretable (31.7%); in Cayenne and Saint-Laurent-du-Maroni combined, 81 of 102 examinations were interpretable (79.4%), and 21 of 102 were technically uninterpretable (20.6%). The overall comparison according to recruitment centre was not statistically significant (global *p* = 0.158). Separate Cayenne and Saint-Laurent-du-Maroni interpretable/uninterpretable counts were not contained in the archived final-analysis output used for this revision and therefore cannot be reconstructed reliably from the manuscript file alone.

### 3.3. Frequency of Uninterpretable Spirometry According to Age

The frequency of technically uninterpretable spirometry differed across age groups. As reported in Table 2, uninterpretable examinations occurred in 13 of 49 children aged 5–7 years, 15 of 49 children aged 8–11 years, and 6 of 45 participants aged 12–18 years.

**Figure 2:**
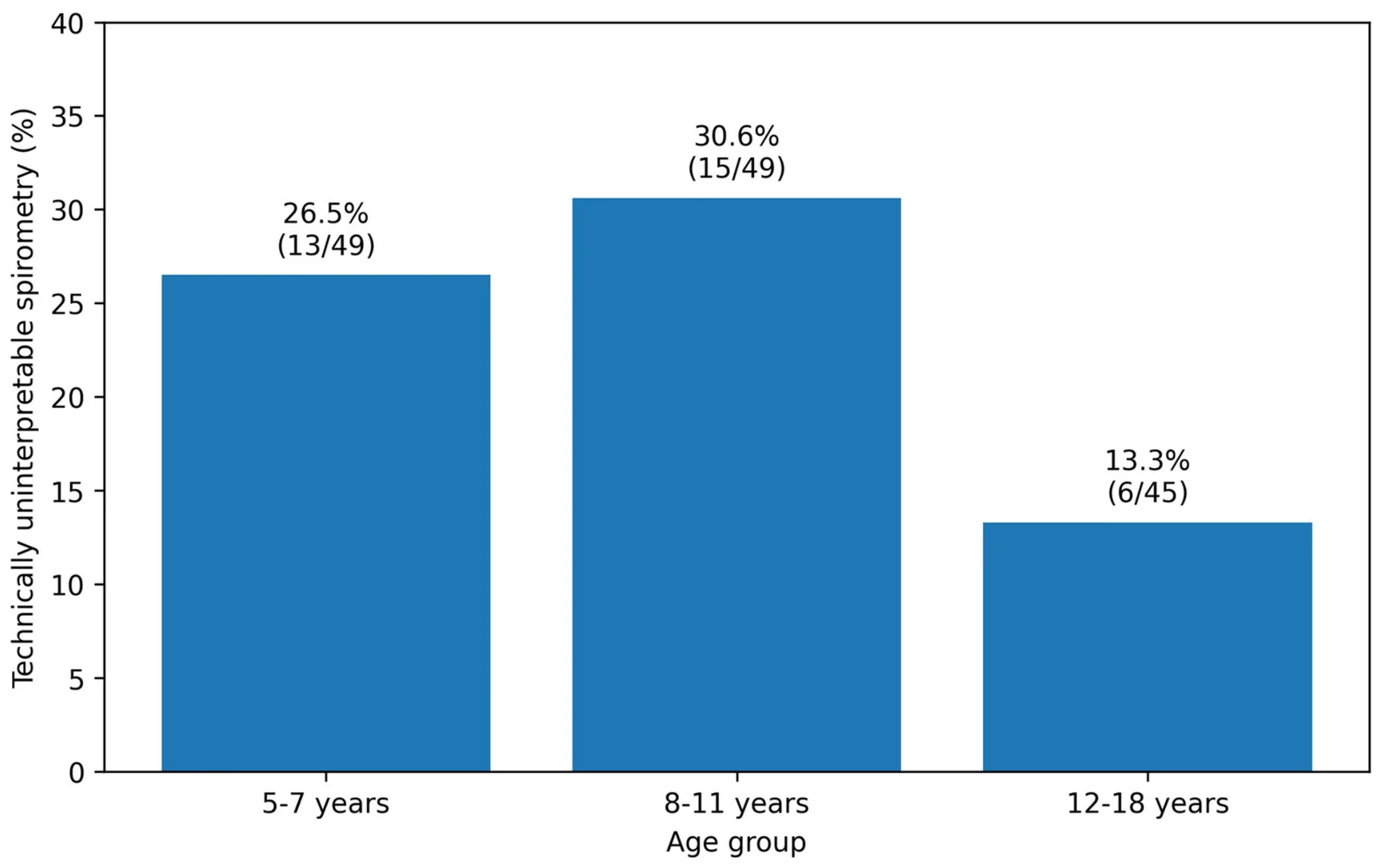
Proportion of technically uninterpretable spirometry according to age group. Data labels show percentage and numerator/denominator. Source: authors’ study data.

The corresponding proportions were 26.5%, 30.6%, and 13.3%, respectively. The lowest proportion was observed among participants aged 12–18 years. However, the pattern was not strictly linear because the frequency was slightly higher in the 8–11-year group than in the 5–7-year group. The distribution of technically uninterpretable spirometry across age categories is illustrated in Figure 2.

#### Graphical Distribution of Uninterpretable Spirometry by Age

As shown in Figure 2, technically uninterpretable spirometry occurred in 13 of 49 children aged 5–7 years (26.5%), 15 of 49 children aged 8–11 years (30.6%), and 6 of 45 participants aged 12–18 years (13.3%). A direct cross-check confirmed that the numerators, denominators, and percentages in Figure 2 are identical to those reported in Table 2; no data inconsistency was identified.

### 3.4. Factors Associated with Technically Uninterpretable Spirometry

The results of the univariable logistic regression analyses are presented in Table 3. Increasing age was significantly associated with a lower likelihood of technically uninterpretable spirometry. Each additional year of age was associated with an approximately 12% reduction in the odds of an uninterpretable examination (OR 0.88, 95% CI 0.78–0.99; *p* = 0.036).

Absence of health insurance was associated with more than twice the odds of uninterpretable spirometry, although the confidence interval was wide and the association was not statistically significant (OR 2.31, 95% CI 0.82–6.53; *p* = 0.114). Children assessed at Kourou also had higher unadjusted odds of uninterpretable spirometry than those assessed at the reference centre, but this association did not reach statistical significance (OR 1.79, 95% CI 0.79–4.04; *p* = 0.161).

Previous acute chest syndrome, tobacco-smoke exposure, eczema, and rural origin were associated with lower point estimates for the odds of uninterpretable spirometry, but none of these associations was statistically significant, as shown in Table 3.

### 3.5. Multivariable Analysis

The parsimonious multivariable logistic regression model included age, sex, recruitment centre, and health-insurance status. The adjusted estimates are presented in Table 4.

After adjustment, the inverse association between age and technically uninterpretable spirometry persisted in direction and magnitude but did not reach conventional statistical significance (adjusted OR 0.89, 95% CI 0.79–1.01; *p* = 0.075). Given the limited number of outcome events, this attenuation may reflect reduced statistical power rather than the absence of an age-related association.

Female sex was not independently associated with uninterpretable spirometry (adjusted OR 1.32, 95% CI 0.58–2.97; *p* = 0.508). Children assessed at Kourou had higher adjusted odds of an uninterpretable examination, but the association remained non-significant (adjusted OR 1.67, 95% CI 0.72–3.88; *p* = 0.230). Similarly, absence of health insurance was associated with an adjusted odds ratio of 1.99, but the estimate was imprecise and not statistically significant (95% CI 0.67–5.88; *p* = 0.214).

Collinearity diagnostics did not indicate problematic overlap among model covariates, and the Hosmer–Lemeshow assessment did not indicate poor calibration. The numerical VIF values and the Hosmer–Lemeshow test statistic, degrees of freedom, and *p*-value were not preserved in the archived exported analysis output and therefore cannot be reported reliably from the available file.

**Figure 3:**
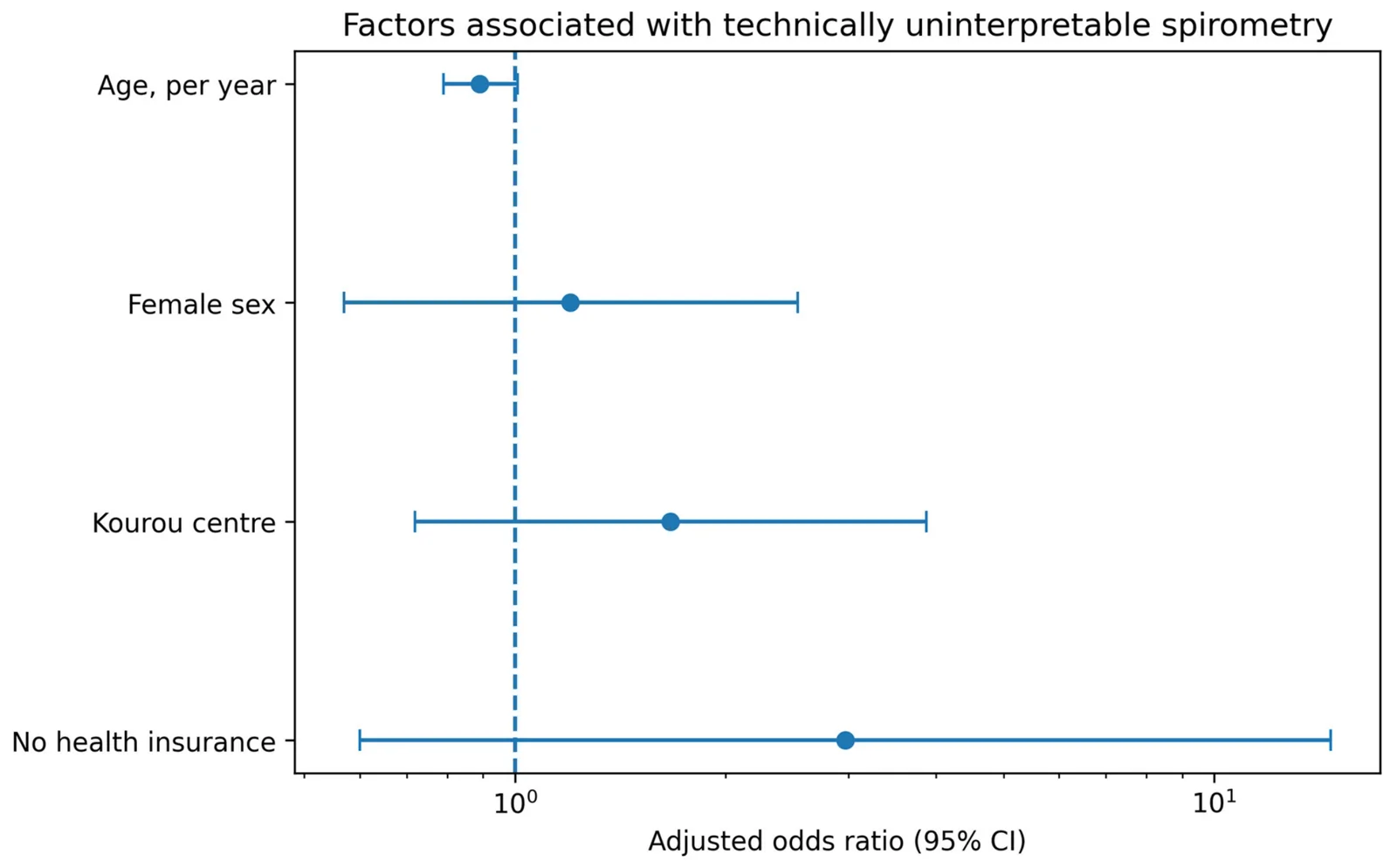
Adjusted odds ratios and 95% confidence intervals for factors associated with technically uninterpretable spirometry.

These adjusted associations are summarized graphically in Figure 3.

### 3.6. Main Findings

In this expanded multicentre cohort, technically uninterpretable spirometry occurred in nearly one-quarter of children undergoing systematic asthma screening. As shown in Table 1, children with uninterpretable examinations were significantly younger than those with interpretable spirometry. The age-group distribution presented in Table 2 and Figure 2 showed that the lowest proportion of uninterpretable examinations occurred among adolescents.

In the univariable analysis presented in Table 3, increasing age was the only factor significantly associated with lower odds of spirometry non-interpretability. In the adjusted model presented in Table 4 and Figure 3, this association was attenuated and no longer reached conventional statistical significance.

## 4. Discussion

### 4.1. Principal Findings

In this expanded multicenter cohort of children with sickle cell disease undergoing systematic asthma screening, technically uninterpretable spirometry occurred in 34 of 143 participants, representing 23.8% of the study population. Children with uninterpretable examinations were significantly younger than those who achieved interpretable spirometry. In univariable analysis, each additional year of age was associated with a 12% reduction in the odds of non-interpretability. This association was attenuated after adjustment for sex, recruitment center, and health-insurance status. No significant independent association was identified for sex, SCD genotype, disease severity, rural residence, hydroxyurea treatment, previous acute chest syndrome, asthma suspicion, tobacco-smoke exposure, rhinitis, or eczema. Although the point estimates suggested higher odds of non-interpretability among children assessed at Kourou and those without health insurance, these associations were imprecise and did not reach statistical significance.

The finding that nearly one-quarter of examinations were technically uninterpretable is clinically relevant. Spirometry is already underused in patients with SCD, including those with asthma or a history of acute chest syndrome [17]. Therefore, failure to obtain an interpretable examination may further reduce access to objective respiratory assessment in a population in whom pulmonary complications are common. Recent data from Ghana also confirm a substantial burden of abnormal spirometric patterns among children with SCD, supporting the importance of obtaining technically reliable measurements whenever respiratory screening is undertaken [18].

### 4.2. Age and Spirometry Interpretability

Younger age was the principal factor associated with technically uninterpretable spirometry. This result is consistent with the developmental requirements of forced expiratory testing. Successful spirometry requires the child to understand several sequential instructions, achieve a complete inspiration, initiate expiration without hesitation, maintain maximal effort, and repeat the maneuver consistently. A pediatric study specifically evaluating the effort required to complete spirometry found that younger children generally required more attempts and greater procedural support than older children and adolescents [19].

The relationship between age and spirometry interpretability was not strictly linear in our cohort, because children aged 5–7 years performed somewhat better than those aged 8–11 years. This finding indicates that chronological age alone does not determine technical success. Attention, motivation, comprehension, previous exposure to pulmonary-function testing, language concordance, operator coaching, and the number of permitted attempts may also influence performance. In the case–control study by Kannan et al., pulmonary-function testing was performed in an SCD cohort with a mean age of 13.5 years and showed better overall feasibility [20]. The older age distribution may partly explain the difference from our findings, although differences in testing protocols, operator expertise, equipment, and quality-control procedures limit direct comparison.

Studies of respiratory screening in children with SCD have shown that symptom questionnaires can be administered even in younger populations, but objective confirmation remains more difficult in preschool-aged children [21,22]. In older children, spirometry may identify respiratory abnormalities that are not detected by questionnaire-based screening alone [21]. Thus, younger age should not be considered a reason to withhold objective testing. Instead, it should identify children who may benefit from longer appointments, age-adapted explanations, visual encouragement, demonstration of the maneuver, and repeat testing by an experienced pediatric operator.

The attenuation of the age association in the adjusted model should also be interpreted cautiously. The adjusted odds ratio remained below one, and the upper boundary of the confidence interval was close to unity. The loss of conventional statistical significance may therefore reflect limited power rather than the complete absence of an age effect. With only 34 outcome events, the study had limited capacity to estimate several independent associations simultaneously. Residual confounding by operator experience, testing duration, language, or prior exposure to spirometry may also have influenced the estimate.

### 4.3. Clinical Importance in Sickle Cell Disease

The high frequency of uninterpretable tests matters because respiratory disease in children with SCD is heterogeneous. Recent spirometry data from African children with SCD demonstrated higher frequencies of restrictive, obstructive, and mixed abnormalities than among children without SCD [18]. Contemporary reviews also emphasize the importance of proactive identification and management of asthma and other pulmonary comorbidities in this population [23]. A failed examination therefore does not represent a neutral result; it leaves uncertainty regarding potentially important respiratory impairment.

Previous multicenter data indicate that diagnostic spirometry remains insufficiently used in people with SCD, even when asthma or acute chest syndrome has been documented [17]. Structured screening questionnaires may improve case identification but cannot replace objective lung-function assessment [21]. Consequently, children whose first spirometry is technically uninterpretable should not be classified as having normal lung function or no asthma. They should remain within the diagnostic pathway until a technically adequate assessment or an appropriate alternative investigation is obtained.

The absence of significant associations with SCD genotype, hydroxyurea treatment, previous acute chest syndrome, or indicators of disease severity suggests that technical interpretability may depend more strongly on developmental and procedural conditions than on the hematologic phenotype itself. However, this finding should not be interpreted as proof that disease-related characteristics have no effect. Severe anemia, fatigue, pain, respiratory limitation, and previous hospital experiences could theoretically impair or facilitate performance in different children. These potentially opposing effects may have reduced measurable associations in the present cohort.

### 4.4. Center-Level and Social Factors

Children assessed at Kourou had higher crude and adjusted point estimates for technically uninterpretable spirometry, although the confidence intervals were wide and included unity. This observation should not be interpreted as evidence of inferior performance at that center. Multicenter differences may reflect variation in the age and clinical characteristics of the children assessed, operator experience, appointment duration, equipment, local workload, language concordance, or completeness of technical documentation.

Spirometry is highly dependent on coaching and real-time recognition of correctable errors. Evidence from home and remote spirometry illustrates this dependency. Unsupervised home spirometry in children and young people with cystic fibrosis was not equivalent to supervised clinic spirometry, emphasizing the importance of direct procedural support and quality control [24]. By contrast, programs combining mobile spirometry with respiratory-therapist training, telehealth support, and standardized review have demonstrated good feasibility in children, including those living in rural or medically underserved areas [25,26,27,28].

French Guiana has a geographically dispersed and multilingual population. Differences in patient-operator language concordance may influence comprehension of the sequential instructions required for forced expiratory maneuvers. Long travel distances, transportation constraints, waiting time, unequal access to specialized respiratory services, and unfamiliarity with pulmonary-function testing may also affect fatigue, anxiety, preparation time, and patient comfort. Although recruitment center and health-insurance status were included as contextual covariates, language proficiency, travel duration, appointment conditions, and patient-operator language concordance were not directly measured; their possible contribution therefore remains exploratory.

Absence of health insurance was associated with approximately twice the odds of an uninterpretable examination, but the estimate was imprecise. Insurance status may function as a proxy for broader vulnerability, including unstable access to care, reduced continuity of follow-up, language barriers, unfamiliarity with medical procedures, or social disadvantage. Because these mechanisms were not measured directly, causal interpretation is not appropriate. Future studies should document preferred language, need for an interpreter, schooling level, previous spirometry experience, travel distance, and the time available for testing.

### 4.5. Alternative Lung-Function Approaches

For children who repeatedly fail conventional spirometry, oscillometry may provide complementary information because it requires tidal breathing rather than a forced expiratory maneuver. Studies in children with asthma or wheeze have shown that oscillometry can be completed successfully and can provide information about bronchodilator responsiveness, particularly when spirometry is difficult [29]. Similar work in children with neuromuscular disease suggests that oscillometry may offer a feasible alternative when forced maneuvers cannot be performed, although it should not automatically be considered interchangeable with spirometry [30].

In practice, an uninterpretable first spirometry should generally lead to a structured second attempt before moving to an alternative test. The repeat pathway could include a separate appointment, preparation through demonstration or video, visual incentive software, instruction in the child’s preferred language, and testing by an operator experienced in pediatric spirometry. Oscillometry, peak-flow variability, specialist assessment, or supervised mobile spirometry could then be considered when conventional spirometry remains unsuccessful.

### 4.6. Reference Equations and the French Guianese Context

Technical interpretability must be distinguished from physiological interpretation. A child may produce technically valid maneuvers while the classification of the resulting values varies according to the reference equation applied. This is particularly relevant in French Guiana because the pediatric population is ethnically and genetically diverse.

Recent pediatric studies have shown that replacing race-specific equations with race-neutral equations can materially alter predicted values and the classification of obstructive, restrictive, or nonspecific spirometric patterns [31,32]. The American Thoracic Society has recommended moving away from race-based correction and considering a race-neutral approach, while acknowledging that interpretation must remain clinically contextualized [33]. Studies in children from The Gambia and in Black and South-East Asian pediatric populations further demonstrate that the choice of equations can alter the proportion classified as having abnormal lung function [34,35].

These issues do not explain technically failed maneuvers, but they are important once a valid examination has been obtained. Future analyses in French Guiana should clearly separate technical quality from the reference-equation-dependent interpretation of FEV_1_, FVC, and FEV_1_/FVC. Sensitivity analyses using race-neutral equations may be particularly informative in this highly admixed population. Broader analyses have shown that the transition to global race-neutral equations may change pulmonary-function interpretation across populations and clinical contexts [36].

### 4.7. Practical Implications

The present findings support a systematic quality-improvement strategy rather than restricting spirometry to older children. First, the exact reason for each unsuccessful examination should be documented, including incomplete inspiration, hesitation, cough, early termination, glottic closure, inconsistent effort, or failure to meet repeatability criteria. Second, the number of attempts, duration of testing, operator, equipment, and need for language assistance should be recorded. Third, children with an unsuccessful first examination should be offered a planned repeat assessment rather than being lost from the screening pathway. In French Guiana, a pragmatic strategy could combine standardized illustrated instructions, short multilingual demonstration videos, age-adapted explanations, experienced pediatric coaching, adequate testing time, periodic harmonized operator training, common quality-control procedures, and planned repeat assessment after an initially unsuccessful test. Centralized or telehealth-supported review of difficult flow-volume curves may further reduce center-level variability.

Gamified or immersive digital preparation, including interactive applications and virtual-reality tools, may improve engagement, reduce anxiety, and help children understand the sequence of forced respiratory maneuvers. Evidence from pediatric rehabilitation, procedural anxiety, asthma education, and pulmonary rehabilitation supports the feasibility of such approaches [37,38,39,40], but direct evidence that they improve spirometry acceptability or repeatability remains limited. Prospective controlled studies should evaluate whether these tools improve first-attempt success, reduce the number of maneuvers and testing time, and increase the proportion of interpretable examinations.

Implementation of contemporary interpretive strategies can be difficult when equipment, software, reference equations, and staff experience vary between centers [41]. A common French Guianese protocol should therefore define technical acceptability, quality grading, bronchodilator procedures, repeat-testing rules, and centralized review arrangements. This approach may reduce intercenter variability while increasing the proportion of children who obtain a definitive functional classification.

### 4.8. Strengths and Limitations

The strengths of this study include its multicenter design, inclusion of the three principal pediatric SCD centers in French Guiana, extension of the initial screening period, and evaluation of an operational outcome with direct clinical consequences. The study also examined multiple demographic, social, hematologic, geographic, respiratory, and atopic factors.

Several limitations should be acknowledged. First, only 34 technically uninterpretable examinations were observed. Despite use of a parsimonious model, this event count limited statistical power and may have resulted in type II error, particularly for age and contextual covariates whose effect estimates remained clinically plausible. Second, operator experience and training may have varied across participating centers and could not be modeled as independent variables because standardized operator-level identifiers and measures of experience were unavailable. Third, the analysis was based on a single retained assessment per participant; consequently, the proportion of children who subsequently achieved acceptable spirometry after additional coaching or a later appointment could not be evaluated. Language concordance, travel time, appointment duration, fatigue, and previous familiarity with spirometry were not systematically recorded. Finally, exact center-specific equipment details and standardized operator-experience measures require confirmation from source records before resubmission.

Taken together, these findings suggest that relatively simple procedural adaptations—including age-adapted preparation, experienced coaching, adequate testing time, and planned repeat assessment—may convert initially uninterpretable examinations into clinically valuable diagnostic information.

## 5. Conclusions

Technically uninterpretable spirometry affected nearly one-quarter of children with SCD undergoing systematic asthma screening in French Guiana. Younger age remained associated with lower test success in direction and magnitude after adjustment, although the study may have lacked sufficient power to demonstrate an independent association conclusively. No statistically significant associations with SCD-related or respiratory characteristics were identified; however, these findings should be interpreted cautiously because the limited number of outcome events reduced the power to detect modest associations. Age-adapted preparation, experienced coaching, planned repeat testing, and harmonization of equipment, operating procedures, operator training, and quality-control practices across Cayenne, Kourou, and Saint-Laurent-du-Maroni may improve the yield and comparability of multicenter respiratory screening in geographically and culturally diverse settings. Simple procedural adaptations may help convert initially uninterpretable examinations into clinically valuable diagnostic information.

## Figures and Tables

**Table 1:** Participant characteristics according to spirometry interpretability. Source: authors’ study data.

Characteristic	Overall, *n* = 143	Interpretable Spirometry, *n* = 109	Uninterpretable Spirometry, *n* = 34	*p*-Value
Age, years, mean ± SD	10.2 ± 3.8	10.5 ± 4.0	8.9 ± 3.0	0.015
Female sex, *n* (%)	78 (54.5)	57 (52.3)	21 (61.8)	0.333
No health insurance, *n* (%)	18 (12.6)	11 (10.1)	7 (20.6)	0.138
HbSS genotype, *n* (%)	108 (75.5)	81 (74.3)	27 (79.4)	0.546
Severe clinical course, >2 admissions, *n* (%)	50 (35.0)	39 (35.8)	11 (32.4)	0.714
Rural origin, *n* (%)	106 (74.1)	82 (75.2)	24 (70.6)	0.590
Kourou centre, *n* (%)	41 (28.7)	28 (25.7)	13 (38.2)	0.158
Hydroxyurea treatment, *n* (%)	110 (76.9)	83 (76.1)	27 (79.4)	0.693
Previous acute chest syndrome, *n* (%)	48 (33.6)	40 (36.7)	8 (23.5)	0.156
Prior asthma suspicion, *n* (%)	36 (25.2)	27 (24.8)	9 (26.5)	0.842
Tobacco-smoke exposure, *n* (%)	23 (16.1)	19 (17.4)	4 (11.8)	0.432
ISAAC Q2–3 positive, *n* (%)	30 (21.0)	23 (21.1)	7 (20.6)	0.949
ISAAC Q4–5 positive, *n* (%)	113 (79.0)	87 (79.8)	26 (76.5)	0.676
Eczema, *n* (%)	20 (14.0)	17 (15.6)	3 (8.8)	0.406
Rhinitis, *n* (%)	115 (80.4)	88 (80.7)	27 (79.4)	0.865

SD: standard deviation; SCD: sickle cell disease; HbSS: homozygous sickle cell anemia; ISAAC: International Study of Asthma and Allergies in Childhood. Percentages are calculated within each column. Source: authors’ study data.

**Table 2:** Frequency of technically uninterpretable spirometry by age group. Source: authors’ study data.

Age Group	Uninterpretable Spirometry, *n*	Total, *n*	Proportion, %
5–7 years	13	49	26.5
8–11 years	15	49	30.6
12–18 years	6	45	13.3

**Table 3:** Univariable logistic regression of factors associated with technically uninterpretable spirometry. Source: authors’ study data.

Variable	Crude OR	95% CI	*p*-Value
Age, per additional year	0.88	0.78–0.99	0.036
Female sex	1.47	0.67–3.24	0.334
No health insurance	2.31	0.82–6.53	0.114
HbSS genotype	1.33	0.52–3.40	0.547
Severe clinical course, >2 admissions	0.86	0.38–1.95	0.715
Rural origin	0.79	0.34–1.86	0.590
Kourou centre	1.79	0.79–4.04	0.161
Hydroxyurea treatment	1.21	0.47–3.10	0.693
Previous acute chest syndrome	0.53	0.22–1.28	0.160
Prior asthma suspicion	1.09	0.45–2.63	0.842
Tobacco-smoke exposure	0.63	0.20–2.00	0.435
ISAAC Q2–3 positive	0.97	0.37–2.51	0.949
ISAAC Q4–5 positive	0.82	0.33–2.06	0.676
Eczema	0.52	0.14–1.91	0.327
Rhinitis	0.92	0.35–2.40	0.865

OR: odds ratio; CI: confidence interval. Source: authors’ study data.

**Table 4:** Multivariable logistic regression of factors associated with technically uninterpretable spirometry. Source: authors’ study data.

Variable	Adjusted OR	95% CI	*p*-Value
Age, per additional year	0.89	0.79–1.01	0.075
Female sex	1.32	0.58–2.97	0.508
Kourou centre	1.67	0.72–3.88	0.230
No health insurance	1.99	0.67–5.88	0.214

## Data Availability

The dataset generated and analyzed during the current study is not publicly available because it contains potentially sensitive clinical information concerning children with sickle cell disease. De-identified data may be made available by the corresponding author upon reasonable request and subject to approval by the responsible institution and compliance with applicable ethical and data-protection regulations.

## Supplementary Materials

The following supporting information can be downloaded at https://www.mdpi.com/article/10.3390/children13091281/s1. File S1: STROBE Statement—Checklist of Items That Should Be Included in Reports of Observational Studies.

## Abbreviations

The following abbreviations are used in this manuscript:

ACS	Acute chest syndrome
ATS	American Thoracic Society
CI	Confidence interval
ERS	European Respiratory Society
FEV_1_	Forced expiratory volume in one second
FVC	Forced vital capacity
ISAAC	International Study of Asthma and Allergies in Childhood
OR	Odds ratio
SCD	Sickle cell disease
SD	Standard deviation
