# Supplementary material for: Factors Associated with Uninterpretable Spirometry During Asthma Screening in Children with Sickle Cell Disease: An Expanded Multicenter Study in French Guiana
Source: Children (Basel). 2026 Sep 21;13(9):1281. doi: 10.3390/children13091281 (PMC13605317; doi:10.3390/children13091281)
Supplement: Supplementary file 1 [file children-13-01281-s001.zip › children-4478931-supplementary.pdf]

Supplementary Material File S1:

STROBE Statement—Checklist of Items That Should Be Included in Reports of Observational Studies

Completed for Manuscript ID: children-4478931

Factors Associated with Uninterpretable Spirometry During Asthma Screening in Children with Sickle Cell Disease: An Expanded Multicenter Study in French Guiana

Study design: prospective multicenter observational study

Note: Page numbers refer to the current 22-page final proof manuscript. Items marked "Not applicable" are not required for this study design; where an item is not explicitly reported, this is stated transparently.

Section	Item	STROBE recommendation	Where/how reported in this manuscript	Page(s)
**Title and abstract**	1(a)	Indicate the study's design with a commonly used term in the title or the abstract.	The Abstract identifies the work as a "prospective multicenter observational study."	p. 1
**Title and abstract**	1(b)	Provide in the abstract an informative and balanced summary of what was done and what was found.	Structured Abstract reports objectives, setting, participants, spirometry-quality definition, statistical approach, main numerical results, and conclusions.	pp. 1–2
**Introduction**	2	Explain the scientific background and rationale for the investigation being reported.	Background describes respiratory morbidity in sickle cell disease, the role and technical limitations of spirometry, pediatric feasibility, and the French Guianese context.	pp. 2–4
**Introduction**	3	State specific objectives, including any prespecified hypotheses.	The final Introduction paragraph states the objective: determine the frequency of technically uninterpretable spirometry and identify demographic, clinical, disease-related, geographic, social, and center-related associated factors.	p. 4
**Methods**	4	Present key elements of study design early in the paper.	The Methods identify a prospective multicenter observational design using an expanded cohort undergoing systematic respiratory assessment.	p. 4
**Methods**	5	Describe the setting, locations, and relevant dates, including periods of recruitment, exposure, follow-up, and data collection.	Recruitment occurred January 2025–May 2026 at Cayenne Hospital, Kourou Hospital, and the Saint-Laurent-du-Maroni site of the University Hospital of French Guiana.	p. 4
**Methods**	6(a)	Give eligibility criteria, and the sources and methods of selection of	Eligibility, clinical stability criteria, exclusions, and the rule retaining	pp. 4–5

Section	Item	STROBE recommendation	Where/how reported in this manuscript	Page(s)
		participants. Describe methods of follow-up where relevant.	one observation per participant are reported. The study uses a single retained screening assessment rather than longitudinal follow-up.	
**Methods**	6(b)	For matched studies, give matching criteria and numbers of exposed and unexposed.	Not applicable: this was not a matched study.	N/A
**Methods**	7	Clearly define all outcomes, exposures, predictors, potential confounders, and effect modifiers. Give diagnostic criteria, if applicable.	The primary outcome (technically uninterpretable spirometry), reconstruction rules, respiratory classification, and candidate explanatory variables are defined in detail.	pp. 6–7
**Methods**	8	For each variable of interest, give sources of data and details of methods of assessment. Describe comparability of assessment methods if there is more than one group.	Respiratory screening, spirometry procedure, ATS/ERS quality criteria, GLI-2012 reference equations, bronchodilator delivery method, and common procedures across the three centers are described. Limitations of archived device metadata are stated.	pp. 5–6
**Methods**	9	Describe any efforts to address potential sources of bias.	Data cleaning, duplicate identification/manual review, source-data verification for uncertain spirometry status, restriction to one observation per participant, common technical criteria, and cautious interpretation of center effects are described.	pp. 6–8
**Methods**	10	Explain how the study size was arrived at.	No formal a priori sample-size calculation is reported. The analysis used the available expanded screening cohort after duplicate removal and eligibility/data-quality checks (212 source records; 143 unique children retained).	pp. 4, 9
**Methods**	11	Explain how quantitative variables were handled in the analyses. If applicable, describe which groupings were chosen and why.	Continuous variables were summarized according to distribution; age was analyzed primarily continuously and secondarily in clinically relevant pediatric groups (5–7, 8–11, and 12–18 years).	pp. 7–8
**Methods**	12(a)	Describe all statistical methods, including those used to control for confounding.	Group comparisons, univariable logistic regression, and a parsimonious multivariable logistic model are described. Age and sex were included a priori; recruitment center and health-insurance status were retained as contextual covariates.	pp. 7–8
**Methods**	12(b)	Describe any methods used to examine subgroups and interactions.	Secondary age-group analyses and center-level comparisons are reported. No formal interaction analysis is reported.	pp. 8, 10–11

Section	Item	STROBE recommendation	Where/how reported in this manuscript	Page(s)
**Methods**	12(c)	Explain how missing data were addressed.	Missing data were not coded as negative responses; the primary multivariable analysis used complete cases.	p. 8
**Methods**	12(d)	If applicable, explain how loss to follow-up was addressed.	Not applicable: the primary analysis was based on one retained screening assessment per participant rather than longitudinal follow-up.	N/A
**Methods**	12(e)	Describe any sensitivity analyses.	No formal sensitivity analysis is reported.	Not reported
**Results**	13(a)	Report numbers of individuals at each stage of the study (e.g., potentially eligible, examined, confirmed eligible, included, and analyzed).	The source database contained 212 records; 69 exact duplicate rows were removed; 143 unique children were retained, including 109 interpretable and 34 technically uninterpretable examinations.	p. 9
**Results**	13(b)	Give reasons for non-participation at each stage.	Duplicate removal is explicitly reported. Other exclusions are defined in Methods, but a numerical breakdown for each exclusion reason is not separately reported in the Results.	pp. 4–5, 9
**Results**	13(c)	Consider use of a flow diagram.	A participant/data-cleaning flow diagram (Figure 1) is provided.	p. 10
**Results**	14(a)	Give characteristics of study participants and information on exposures and potential confounders.	Participant characteristics by spirometry interpretability are presented in Table 1, including age, sex, insurance, genotype, disease severity, rural origin, center, hydroxyurea, ACS history, asthma suspicion, smoke exposure, ISAAC responses, eczema, and rhinitis.	pp. 10–11
**Results**	14(b)	Indicate the number of participants with missing data for each variable of interest.	A separate variable-by-variable missing-data count is not presented; the Methods state that missing data were not coded as negative and that complete cases were used for the primary multivariable model.	p. 8
**Results**	14(c)	Summarize follow-up time, if relevant to a cohort study.	Not applicable to the primary analysis, which uses one retained screening assessment per participant.	N/A
**Results**	15	Report numbers of outcome events or summary measures over time.	Technically uninterpretable spirometry occurred in 34/143 participants (23.8%); 109/143 examinations were interpretable. Age-specific frequencies are also presented.	pp. 9–12
**Results**	16(a)	Give unadjusted estimates and, if applicable, confounder-adjusted estimates and their precision. Make clear which confounders were adjusted for and why.	Table 3 reports crude ORs and 95% CIs; Table 4 reports adjusted ORs and 95% CIs. The adjustment set (age, sex, recruitment center, health-insurance status) and rationale are stated in Methods.	pp. 12–14

Section	Item	STROBE recommendation	Where/how reported in this manuscript	Page(s)
**Results**	16(b)	Report category boundaries when continuous variables were categorized.	Age categories are explicitly defined as 5–7, 8–11, and 12–18 years.	pp. 8, 11–12
**Results**	16(c)	If relevant, consider translating estimates of relative risk into absolute risk for a meaningful time period.	Not applicable: the outcome is cross-sectional technical interpretability at the screening examination, not a time-to-event risk estimate.	N/A
**Results**	17	Report other analyses done—e.g., subgroup and interaction analyses, and sensitivity analyses.	Age-group frequencies, center-level comparison, univariable analyses, adjusted analysis, and graphical adjusted estimates are reported. No formal interaction or sensitivity analyses are reported.	pp. 11–14
**Discussion**	18	Summarize key results with reference to study objectives.	The Principal Findings section summarizes the 23.8% uninterpretable rate, younger age association, attenuation after adjustment, and lack of significant independent associations for other evaluated factors.	pp. 14–15
**Discussion**	19	Discuss limitations of the study, taking into account sources of potential bias or imprecision. Discuss both direction and magnitude of any potential bias.	A dedicated Strengths and Limitations section discusses the limited number of events, possible type II error, unavailable operator-level variables, single retained assessment, unmeasured language/travel/testing factors, and incomplete center-specific equipment metadata.	pp. 18–19
**Discussion**	20	Give a cautious overall interpretation of results considering objectives, limitations, multiplicity of analyses, results from similar studies, and other relevant evidence.	The Discussion interprets the age finding cautiously, notes limited power and residual confounding, compares findings with prior pediatric/SCD literature, and avoids causal interpretation of center and insurance associations.	pp. 14–19
**Discussion**	21	Discuss the generalisability (external validity) of the study results.	The Discussion addresses the geographically dispersed, multicultural and multilingual French Guianese setting and discusses implications for multicenter respiratory screening and harmonization across centers.	pp. 16–19
**Other information**	22	Give the source of funding and the role of the funders for the present study and, if applicable, for the original study on which the present article is based.	The manuscript states: “This research received no specific grant from any funding agency in the public, commercial, or not-for-profit sectors.”	p. 19

Additional reporting information in the manuscript

Ethics and data protection: the manuscript includes an Institutional Review Board Statement, Informed Consent Statement, Data Availability Statement, declaration of artificial-intelligence use, and Conflict of Interest statement (pp. 19–20). These items are not separate items in the core 22-item STROBE checklist but are reported for journal compliance.
